# Clozapine and Risk of Haematological Malignancies: Insights from a Meta-Analysis

**DOI:** 10.1192/bjo.2025.10132

**Published:** 2025-06-20

**Authors:** Omar Kassar, Osama Hassan, Moaz Elsayed Abouelmagd, Muataz Kashbour, Omar Shaheen

**Affiliations:** ^1^Faculty of Medicine, Alexandria University, Alexandria, Egypt; ^2^Medical Research Group of Egypt, Negida Academy, Arlington, Massachusetts, USA; ^3^Central and North West London NHS Foundation Trust, London, United Kingdom; ^4^Faculty of Medicine, Zagazig University, Zagazig, Egypt; ^5^Faculty of Medicine, Cairo University, Cairo, Egypt; ^6^Dignostic Radiology Department, National Cancer Institute, Misrata, Libya

## Abstract

**Aims:** Clozapine, the first and most effective atypical antipsychotic for schizophrenia, is typically reserved for treatment-resistant cases due to its serious adverse effects. Recent studies have linked clozapine to an increased risk of haematological malignancies (HM). This meta-analysis is the first to systematically investigate this association.

**Methods:** We performed this study in accordance with the Cochrane Handbook for Systematic Reviews and Meta-analysis of Interventions. Eligible studies included involving patients treated with clozapine, regardless of the primary psychiatric diagnosis, cohort, case-control, cross-sectional that reported the association between clozapine use and the risk of haematological malignancies.

**Results:** Five studies were included in our meta-analysis (three retrospective cohorts and two case-control studies) involving a total of 211,427 patients. The overall odds of developing HM were significantly higher in the clozapine group compared with the control group (OR=2.1, 95% CI, 1.39–3.18, p=0.00005, I2=83%). Sensitivity analysis was conducted, and heterogeneity was resolved by exclusion of the study by Brainerd et al. The overall effect after its exclusion suggested a significant increase in the odds of HM (OR=2.45, 95% CI, 1.75–3.43, p< 0.00001, I²=48%). Subgroup analysis showed that the odds of developing leukaemia (OR=4.02, 95% CI, 2.22–7.27, p<0.00001), and lymphoma (OR=6.27, 95% CI, 2.83–13.9, p<0.00001) were significantly higher than the combined risk for all HM. There was no significant association between clozapine and HM with a cumulative dose of <999 defined daily doses (DDD) (OR=1.11, 95% CI, 0.85–1.46, p=0.44) or 1000–2999 DDD (OR=1.34, 95% CI, 0.76–2.34, p=0.31). However, for patients with a cumulative dose of 3000–4999 DDD and >5000 DDD, the risk of HM was significantly higher in the clozapine group (OR=2.04, 95% CI, 1.46–2.86, p<0.0001), (OR=2.45, 95% CI, 1.32–4.48, p=0.004), respectively. The association between clozapine and haematological malignancies became statistically significant after 5 years of follow-up (OR=2.32, 95% CI, 1.5–3.59, p=0.0002).

**Conclusion:** Despite the increased risk of HM, clozapine treatment in schizophrenia patients is associated with a significantly lower long-term all-cause mortality rate compared with other antipsychotic use. The small risk should not deter its use or not fuel “clozapine-phobia”. The clinical implication of our study is to raise awareness among the psychiatrists about this risk. Haematological abnormalities could be interpreted as typical adverse effects of clozapine, leading to diagnostic bias and delays in malignancy diagnosis.

